# Prevalence of cervical anterior and posterior spondylolisthesis and its association with degenerative cervical myelopathy in a general population

**DOI:** 10.1038/s41598-020-67239-4

**Published:** 2020-06-26

**Authors:** Kimihide Murakami, Keiji Nagata, Hiroshi Hashizume, Hiroyuki Oka, Shigeyuki Muraki, Yuyu Ishimoto, Munehito Yoshida, Sakae Tanaka, Akihito Minamide, Yukihiro Nakagawa, Noriko Yoshimura, Hiroshi Yamada

**Affiliations:** 1 Department of Orthopaedic Surgery, Wakayama Medical University, 811-1 Kimiidera, Wakayama City, Wakayama, Japan; 2 Department of Medical Research and Management for Musculoskeletal Pain, 22nd Century Medical and Research Center, Faculty of Medicine, The University of Tokyo, 7-3-1 Hongo, Bunkyo-ku, Tokyo, Japan; 3 Department of Preventive Medicine for Locomotive Organ Disorders, 22nd Century Medical and Research Center, Faculty of Medicine, The University of Tokyo, 7-3-1 Hongo, Bunkyo-ku, Tokyo, Japan; 4 Department of Orthopaedic Surgery, Sumiya Orthopaedic Hospital, 337 Yoshida, Wakayama City, Wakayama, Japan; 5 Department of Orthopaedic Surgery, Faculty of Medicine, The University of Tokyo, 7-3-1 Hongo, Bunkyo-ku, Tokyo, Japan; 6 Department of Orthopaedic Surgery, Wakayama Medical University Kihoku Hospital, 219 Myoji, Katsuragi Town, Ito County, Wakayama, Japan

**Keywords:** Diseases, Risk factors

## Abstract

The purpose of this study was to examine the prevalence of cervical spondylolisthesis according to age and vertebral level and its association with degenerative cervical myelopathy (DCM). This study included 959 participants (319 men and 640 women; mean age, 66.4 years) in the Wakayama Spine Study from 2008 to 2010. The outcome measures were cervical spinal canal (CSC) diameter at C5 level on plain radiographs, the degree of cervical spondylosis using the Kellgren-Lawrence (KL) grade, cervical cord compression on sagittal T2-weighted magnetic resonance imaging, and physical signs related to DCM. The prevalence of cervical anterior and posterior spondylolisthesis was investigated in men and women by age. In addition, logistic regression analysis determined the association between CSC diameter, posterior spondylolisthesis, and clinical DCM after overall adjustment for age, sex, and body mass index. The prevalence of anterior spondylolisthesis was 6.0% in men and 6.3% in women, and that of posterior spondylolisthesis was 13.2% and 8.9%, respectively. In addition, posterior spondylolisthesis prevalence increased with age in both sexes. Logistic regression analysis revealed that developmental canal stenosis (≤13 mm) and cervical posterior spondylolisthesis are independent significant predictive factors for DCM. The prevalence of degenerative cervical posterior spondylolisthesis was increasing with age and more frequent in men than in women. Narrow canal and degenerative cervical posterior spondylolisthesis on X-ray may be useful in predicting or diagnosing DCM.

## Introduction

Degenerative cervical myelopathy (DCM) is a disease concept that includes age-related degenerative changes of the cervical spine, such as intervertebral disc disease, vertebral remodeling, hypertrophy and/or ossification of spinal ligaments, and spondylolisthesis. Cervical spondylolisthesis prevalence has been reported as low as 5.2% to 12%^1–3^, whereas that of lumbar spondylolisthesis is 15.8% to 19.7%^4,5^. Thus, cervical spondylolisthesis has received less attention than lumbar spondylolisthesis.

Cervical anterior spondylolisthesis (AS) and posterior spondylolisthesis (PS) are mainly caused by degeneration of disc and facet joint^6^. Cervical spondylolisthesis results in not only cervical pain but also radiculopathy or myelopathy as it progresses and thus should never be neglected. In the aging society, the number of patients with degenerative changes of the cervical spine is expected to increase. Nevertheless, currently, reports on the association between cervical spondylolisthesis and DCM are few. Cervical spondylolisthesis could be relatively easily diagnosed by cervical plain X-ray (lateral view). If the association between cervical spondylolisthesis and DCM become clear, a risk assessment of DCM could be expected in patients diagnosed as having cervical spondylolisthesis using relatively easy-to-use imaging tests, such as plain radiographs.

Hence, this study aimed to examine the prevalence of cervical spondylolisthesis according to sex, age and vertebral level and its association with DCM.

## Methods

### Compliance with ethical standards

This study was conducted in accordance with the Declaration of Helsinki and the study design was approved by the Ethics Committee of the Wakayama Medical University. All volunteers provided written informed consent for participation.

### Participants

This cross-sectional observational study was approved by the appropriate ethics committee. All participants provided written informed consent, and the study design was approved by the appropriate ethics review boards. This study is a part of “The Wakayama Spine Study: a population-based cohort,” which is a large-scale population-based magnetic resonance imaging (MRI) study. Details of The Wakayama Spine Study have been described elsewhere^7,8^. Briefly, the baseline survey of The Wakayama Spine Study was conducted between 2008 and 2010 in a mountainous region in Hidakagawa, Wakayama, and a coastal region in Taiji, Wakayama. A total of 1063 residents of the Hidakagawa, Taiji region were recruited for MRI examination, of which 52 people declined. Thus, 1011 inhabitants were enrolled in this study. Of the 1011 participants, those with MRI-sensitive implantable devices (such as pacemakers) and other unqualified individuals were excluded. Subsequently, the cervical spine of 985 individuals was scanned with MRI. Four participants who had undergone previous cervical surgery and another four with poor image quality were excluded from the analysis. A total of 977 participants were included in the final analysis. Radiological evaluation of the cervical vertebrae was also performed in 959 subjects. The results of both MRI and radiography were available for 959 participants (males, 319; females, 640) aged 21 to 97 years (average, 67.3 years; female 65.9 years).

Physical measurements included height (meter), body weight (kilogram), and body mass index (BMI; body weight [kilogram] / height^2^ [m^2^]). Medical information on sensory disorder, Hoffman reflex, Babinski reflex, and deep tendon reflex of the patellar tendon was obtained by an experienced orthopedic surgeon. Hoffmann reflex was tested in a neutral position by flicking the distal phalange of the middle finger and observing whether bending of the distal phalanx of the thumb occurs^9^. The Babinski reflex was elicited by firmly sweeping from the lateral part of the sole to the base of the toes with the tip of a reflex hammer and observing the hallux extensor response^10^.

### Radiographic measures

**Figure 1:**
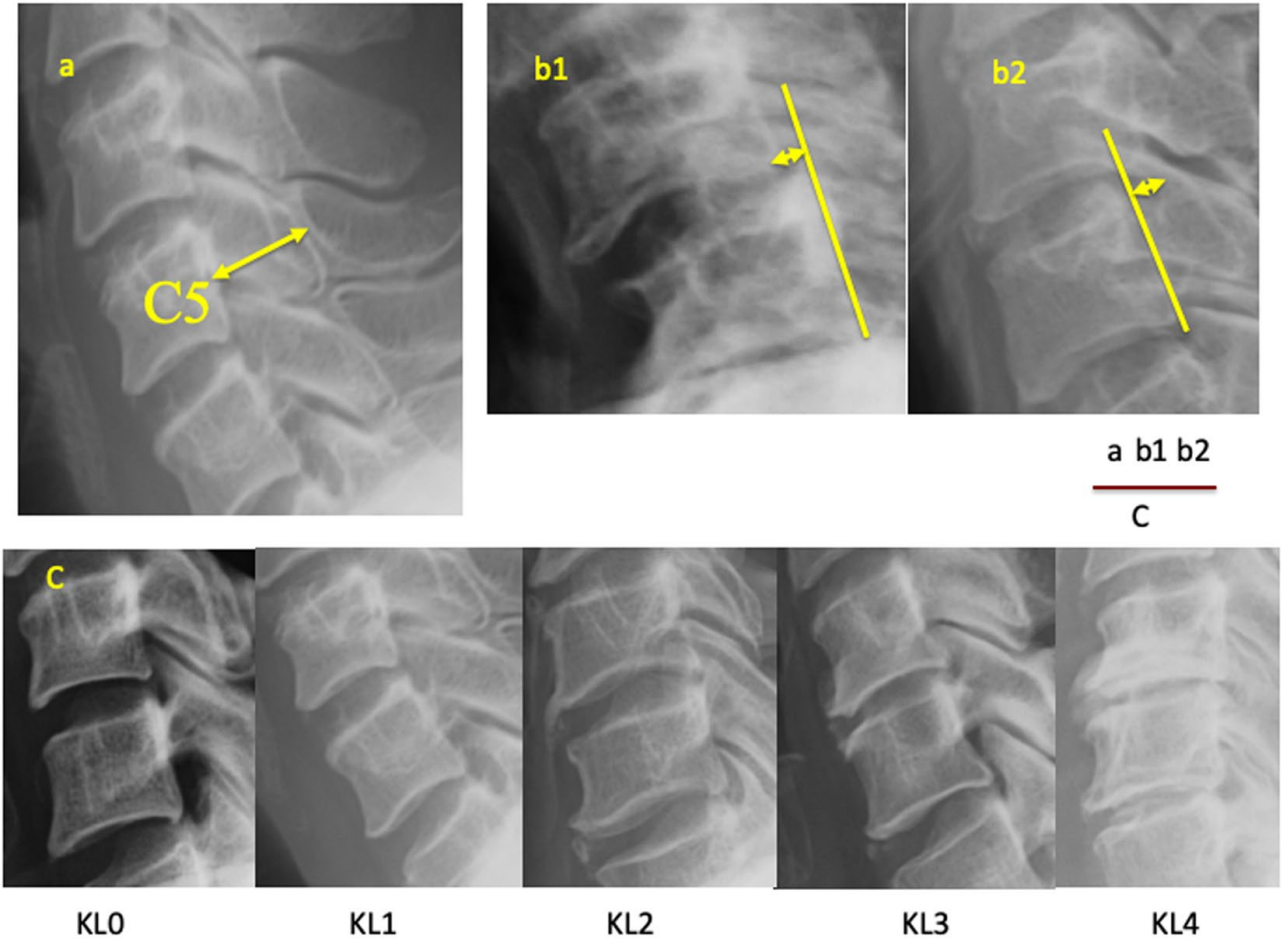
Radiographic measurements of the cervical spine. (**a**) Diameter of the cervical spinal canal at C5. (**b**) Measurement of cervical anterior/posterior spondylolisthesis. (**c**) Kellgren-Lawrence classification for grading of cervical spondylosis.

All subjects underwent lateral radiograph with their neck in the neutral position. Radiographic information, which was put on film, was evaluated and calibrated using the ruler. Sagittal spinal canal diameter at C5 level was measured as the shortest distance from the midpoint between the vertebral body’s superior and inferior endplates to the spinolaminar line^11^. Slippage distance was measured as the distance from the posteroinferior corner of the cranial vertebral body to the tangential line along the posterior border of the caudal vertebral body^12^. We defined the spondylolisthesis group as those with ≥2 mm of slippage on the baseline lateral radiographs. The degree of cervical spondylosis was determined using the Kellgren-Lawrence (KL) grade as follows: KL0, normal; KL1, slight osteophytes; KL2, definite osteophytes; KL3, disc space narrowing with osteophytes; KL4, bone sclerosis, disc space narrowing, and large osteophytes (Fig. 1).

### MRI

An MRI scan of the cervical spine was obtained for each participant using a 1.5-T Excelart imaging system (Toshiba, Tokyo, Japan). All scans were performed in the supine position, except for participants with a rounded back, who used a triangular pillow under their heads and knees. The imaging protocol included a sagittal T2-weighted fast spin-echo pulse sequence (repetition time, 4000 ms; echo time, 120 ms; field of view, 300 × 320 mm) and an axial T2-weighted fast spin-echo pulse sequence (repetition time, 4000 ms; echo time, 120 ms; field of view, 180 × 180 mm)^7,8^.

### Definition of clinical DCM

Physical findings concerning sensory disturbances, Hoffmann reflex, Babinski reflex, and deep tendon reflex of the patellar tendon were examined by an experienced orthopedic surgeon. A myelopathic sign was defined as the presence of patellar tendon hyperreflexia, Hoffmann reflex, or Babinski reflex. Myelopathy was defined clinically by the presence of myelopathic signs (e.g., Hoffmann reflex), which was usually accompanied by bilateral sensory deficits, or poor bowel/bladder function. Among the participants with myelopathic signs, cervical cord compression was the essential condition for diagnosing DCM^13,14^.

### Statistical analysis

Baseline characteristics were compared between sexes using Student’s *t*-test. Cochran-Armitage trend test was used to evaluate the association of cervical spinal canal (CSC) diameter, KL grade, and spondylolisthesis prevalence with aging. Prevalence of KL grades ≥3 and ≥4 and cervical AS and PS was compared between participants with and those without DCM using the chi-square test. Age, BMI, and CSC diameter were compared between participants with and those without DCM using non-paired Student’s *t*-test. To determine the association of DCM with radiographic factors, logistic regression analysis was employed after overall adjustment for age, sex, and BMI. All statistical tests were performed at a significance level of 0.05 (two-sided). Data were analyzed using JMP PRO version 14 (SAS Institute, Inc., Cary, NC, USA).

## Results

### Characteristics of the participants

Baseline characteristics of the 959 participants (men, 319; women, 640), including anthropometric measurements and physical performance data, are listed in Table 1. No significant difference in age between sexes was found. Height, weight, BMI, and grip strength were significantly higher in men than in women (p < 0.001).

**Table 1:** Characteristics in men and women.

	Men	Women
N	319	640
Age	67.3 ± 13.8	65.9 ± 13.3
Height, cm	164.6 ± 7.2***	151.6 ± 7.2
Weight, kg	64.4 ± 11.6***	53.0 ± 9.4
Body mass index, kg/m^2^	23.7 ± 3.4**	23.1 ± 3.7
Grip strength, kg	37.9 ± 9.1***	23.9 ± 5.9

Significantly different from women by Student’s t-test (*p < 0.05, **p < 0.01, ***p < 0.001).

Values are the mean ± standard deviation (SD). N = number.

### Sex and age differences in AS and PS

Table 2 shows the age-related differences in AS and PS on radiograph in men and women among different age groups. AS prevalence was most frequently observed at C4 levels, while PS prevalence was mostly noted at C4 and C5 levels in both sexes.

**Table 2:** Prevalence of cervical anterior (**A**) and posterior (**B**) spondylolisthesis in each vertebral level by sex and age.

Age strata	C2	C3	C4	C5	C6	C7
**A**						
*Men*						
Overall (N = 319)	1.6	2.5	3.1	0.9	0.3	0.6
<50 (N = 36)	2.8	2.8	0	0	0	0
50–59 (N = 57)	0	0	0	0	0	0
60–69 (N = 65)	4.6	1.5	1.5	0	0	1.5
70–79 (N = 89)	1.1	4.5	5.6	1.1	0	1.1
≥80 (N = 72)	0	2.8	5.6	2.8	1.4	0
*Women*						
Overall (N = 640)	0.9	3	4.8	1.4	1.1	0
<50 (N = 86)	2.3	2.3	5.8	1.2	0	0
50–59 (N = 116)	0.9	3.4	3.4	0.9	0.9	0
60–69 (N = 158)	0.6	1.3	3.8	1.9	1.3	0
70–79 (N = 165)	0	4.2	5.5	1.8	2.4	0
≥80 (N = 115)	1.7	3.5	6.1	0.9	0	0
**B**						
*Men*						
Overall (N = 319)	0	6.9	10	9.1	1.6	0
<50 (N = 36)	0	2.8	2.8	5.6	0	0
50–59 (N = 57)	0	3.5	5.3	5.3	0	0
60–69 (N = 65)	0	13.8	18.5	12.3	0	0
70–79 (N = 89)	0	6.7	10.1	11.2	2.2	0
≥80 (N = 72)	0	5.6	9.7	8.3	4.2	0
*Women*						
Overall (N = 640)	0.3	2.2	4.8	6.3	0.6	0
<50 (N = 86)	0	0	0	1.2	0	0
50–59 (N = 116)	0.9	1.7	5.2	9.5	0	0
60–69 (N = 158)	0	1.9	5.1	7.6	0	0
70–79 (N = 165)	0	4.2	5.5	6.1	1.8	0
≥80 (N = 115)	0.9	1.7	7	5.2	0.9	0

**(B**) N = number. Values are percentages for each vertebral level.

### Radiographic measures stratified by sex and age

Table 3 shows the age-related differences in CSC diameter, KL grade, and AS and PS prevalence on radiograph in men and women among different age groups. CSC diameter was significantly narrower with age in women, and the CSC diameter of women was narrower than that of men in each age group. The number of spondylosis of KL ≥ 3 (p < 0.001) and KL ≥ 4 (p < 0.001) was increasing with age. PS prevalence increased with age in men (p = 0.047) and women (p = 0.029).

**Table 3:** Radiographic measures of cervical spine stratified by sex and age strata.

Radiographic measures					
Age strata	Diameter of cervical spinal canal (mm)	KL grade % (N)		Anterior spondylolisthesis	Posterior spondylolisthesis
		≥3	≥4	(%)	(%)
*Men*					
Overall	14.8 ± 1.3	80.3 (256)	53.9 (172)	6	13.2
<50	15.2 ± 1.3	38.9 (14)	16.7 (6)	0	5.6
50–59	14.8 ± 1.7	80.7 (46)	38.6 (22)	0	8.8
60–69	14.9 ± 1.2	81.5 (53)	52.3 (34)	6.2	12.3
70–79	14.8 ± 1.2	85.4 (76)	67.4 (60)	10.1	18
≥80	14.4 ± 1.1	93.1 (67)	69.4 (50)	8.3	15.3
*Women*					
Overall	14.1 ± 1.2	79.7 (510)	48.1 (308)	6.3	8.9
<50	14.5 ± 1.3	52.3 (45)	17.4 (15)	3.5	1.2
50–59	14.4 ± 1.3	78.4 (91)	41.4 (48)	2.6	9.5
60–69	14.1 ± 1.1	79.7 (126)	51.3 (81)	4.4	9.5
70–79	13.9 ± 1.1	86.1 (142)	57.6 (95)	9.7	10.3
≥80	13.8 ± 1.0	92.2 (106)	60.0 (69)	9.6	11.3

N = number.

Otherwise indicated, values are mean ± standard deviation (SD) for each age strata in men and women.

### Association between cervical anterior/posterior spondylolisthesis and DCM

The association between the radiographic measurement of cervical spine and DCM is shown in Table 4. Age in both men and women was not significantly associated with DCM (p = 0.67 in men, p = 0.06 in women). Similarly, BMI showed no association with DCM. CSC diameter in men was not significantly associated with DCM (p = 0.11), whereas that in women was significantly associated with DCM (p < 0.0001).

**Table 4:** Association between radiographic measures of cervical spine and degenerative cervical myelopathy.

	DCM (−)	DCM (+)	P-value
*Men*			
N	316	3	
Age, y	67.4 ± 0.8	64.0 ± 8.0	0.67
Body mass index, kg/m^2^	23.7 ± 0.2	20.5 ± 2.0	0.11
Diameter of cervical spinal canal	14.8 ± 0.1	13.5 ± 0.8	0.11
KL grade (≥3) % (N)	80.1 (253)	100 (3)	0.49
KL grade (≥4) % (N)	54.1 (171)	33.3 (1)	0.39
Anterior spondylolisthesis, % (N)	6.0 (19)	0 (0)	0.66
Posterior spondylolisthesis, % (N)	12.3 (39)	100 (3)	<.0001
*Women*			
N	617	23	
Age, y	65.7 ± 0.5	71.1 ± 2.8	0.06
Body mass index, kg/m^2^	23.0 ± 0.1	24.4 ± 0.8	0.08
Diameter of cervical spinal canal	14.1 ± 0.04	13.1 ± 0.2	<0.0001
KL grade (≥3)	79.4 (490)	87.0 (20)	0.98
KL grade (≥4)	47.5 (293)	65.2 (15)	0.21
Anterior spondylolisthesis, % (N)	6.3 (39)	4.4 (1)	0.7
Posterior spondylolisthesis, % (N)	8.4 (52)	21.7 (5)	0.03

DCM = degenerative cervical myelopathy, N = number.

Except where otherwise indicated, values are the mean ± SD.

For continuous outcomes, comparison was by the Student’s t-test.

For categorical outcomes, comparison was by the chi-square test.

Spondylosis of KL ≥ 3 in both men and women was not significantly associated with DCM (p = 0.49 in men, p = 0.98 in women), and spondylosis of KL ≥ 4 was also not significantly associated with DCM in both sexes (p = 0.39 in men, p = 0.21 in women). Moreover, AS was not significantly associated with DCM in men (p = 0.66) and women (p = 0.70), whereas PS was significantly associated with DCM in men (p < 0.0001) and women (p = 0.03).

### Multiple logistic regression analysis of radiographic measures of cervical spine for DCM

Furthermore, multiple logistic regression analysis was performed to evaluate the predictive factors for DCM in radiographic measures after adjustment for age, sex, and BMI (Table 5). CSC diameter (odds ratio [OR], 2.4; 95% confidence interval [CI], 1.6–3.6; p < 0.0001) and cervical PS (OR, 4.3; 95% CI, 1.7–10.1; p = 0.0011) were independent significant predictive factors for DCM. In addition, concurrence of developmental canal stenosis (≤13 mm) and cervical PS was a more significant predictive factor for DCM (OR, 19.7; 95% CI, 5.6–63.1; p < 0.0001).

**Table 5:** The multiple logistic regression analysis of radiographic measures of cervical spine for degenerative cervical myelopathy.

	DCM	
	OR (95%CI)	P-value
Diameter of cervical spinal canal, mm (−1 mm)	2.4 (1.6–3.6)	<0.0001
Cervical posterior spondylolisthesis	4.3 (1.7–10.1)	0.0011
Developmental canal stenosis (13 mm or less) and cervical posterior spondylolisthesis	19.7 (5.6–63.1)	<0.0001

^†^OR was calculated by multiple logistic regression analysis after adjustment for age, gender, and body mass index.

DCM = degenerative cervical myelopathy, OR = odds ratio, CI = confident interval.

## Discussion

This study examined the relationship between radiographic measures of cervical spine and DCM. We found that CSC diameter on plain X-rays and cervical PS are factors significantly related to DCM in the general population.

The prevalence of AS is 6.0% in men and 6.3% in women, while that of PS is 13.2% in men and 8.9% in women; both conditions are more common in the elderly. AS most often occurs at C4 and PS at C4 or C5 in both men and women. In our study, AS was not significantly related to DCM in either men or women; however, PS was significantly related to DCM in both sexes. Moreover, this study found no relationship between cervical spondylosis (i.e., KL grade 3 or 4) and DCM. One reason why cervical spondylosis and DCM are not related is the differences in the anteroposterior diameter of the vertebral canal, i.e., participants without myelopathy in our study had a larger anteroposterior diameter of the vertebral canal than those with myelopathy. In addition, relative retention of the anteroposterior diameter of the vertebral canal despite osteophyte formation or intervertebral disc degeneration could be a reason why myelopathy did not develop^15^.

A previous study found that both AS and PS often occur at C3 and C4. Our study found that AS often occurs at C4 and PS often occurs at C4 or C5; these findings are similar to the results of previous studies^16^. Vertebral displacement or slippage, which results from intervertebral disc degeneration in the more mobile lower cervical spine, mainly occurs at the mid-cervical level with osteophyte formation and thickening of the facet joints. Degenerative changes reduce lower cervical spine mobility; thus, the load on adjacent vertebrae increases to compensate^17^. Teraguchi *et al*.^18^ showed that intervertebral disc degeneration occurs most frequently at C5–C6 in the cervical spine. Reduced lower cervical spine mobility associated with degeneration could cause vertebral displacement or slippage at the mid-cervical level. Nevertheless, AS and PS may not necessarily occur via the same mechanism. Studies reported that the facet joints normally serve as restraints against spondylolisthesis; however, facet joint degeneration destroys those restraints, thereby causing spondylolisthesis^19,20^. Moreover, Jun *et al*.^21^ reported that T1 slope is significantly larger in the degenerative cervical spondylolisthesis group than in the control group. They suggested that a high T1 slope may be a predisposing factor for the development of degenerative cervical spondylolisthesis. Hence, disc degeneration, T1 slope, and facet degeneration are possible causes of degenerative cervical AS.

Based on the tilting or sliding of vertebral bodies as observed during flexion to extension, Kokubun *et al*.^22^ surmised that sliding is predominant in PS. In addition, Lee *et al*.^23^ suggested that intervertebral disc space narrowing causes cervical PS.

Furthermore, facet joint degeneration has been cited as a cause of PS, and PS increased with age and was more prevalent in men than in women (13.2% vs. 8.9%). Uhrenholt *et al*.^24^ reported that degenerative changes affecting the cartilage, osteocartilaginous junction, and subchondral bone of the facet joints are significantly related to age and are more severe in men. Ndubuisi *et al*.^25^ suggested that changes in the cervical spine differ between sexes because men and women performed different types of work in rural areas. For example, men may perform work involving heavy loads, such as farming, while women may do work involving lighter loads, such as household chores and cooking. In our study, the subjects were from fishing and rural communities where men performed work involving a relatively heavy load, which in turn could explain why PS was more prevalent in men.

In this study, no relationship between AS and DCM was found; however, a relationship between PS and DCM was observed. The pincer mechanism reported by Penning *et al*.^26^ plays a substantial role in the relationship between PS due to intervertebral disc degeneration and DCM. During extension of the neck, the spinal cord is pinched between the postero-inferior margin of the superior vertebral body and the antero-superior margin of the lamina of the inferior vertebra, which has been found to result in dynamic spinal cord compression. Thus, PS may be a dynamic factor for DCM. In addition, a previous study reported that ligamentum flavum buckling could lead to spinal stenosis^22^. Satomi *et al*.^27^ stated that dynamic spinal cord compression between the postero-inferior margin of a vertebral body and the lamina of a superior vertebra resulting from AS during anteflexion is associated with the development of myelopathy. In addition, AS was noted in three participants with displacement of ≥3.5 mm; however, no patient developed DCM. Hence, AS may not necessarily be a mechanism involved in myelopathy. Myelopathy was also less likely to develop because ligament buckling did not occur in AS.

A similar study by Yukawa *et al*.^28^ compared the anteroposterior diameter of the vertebral canal by sex and reported that women had a vertebral canal with a small anteroposterior diameter. Nagata *et al*.^7^ reported that DCM was more likely to develop as a result of a vertebral canal with a small anteroposterior diameter. These results indicate that women have a higher risk of developing DCM. Moreover, current results revealed that a combination of developmental spinal stenosis and PS would lead to an even higher risk of developing DCM.

The key aspect of this study is that parameters, such as the CSC diameter and cervical PS on plain X-rays of the cervical spine, could be used to predict the risk of DCM. These parameters may facilitate screening for DCM.

This study has several limitations. First, although this study included more than 1000 participants, the participants may not represent the general population because they were recruited from only two areas in Japan. Nonetheless, anthropometric measurements were compared between this study’s participants and the general Japanese population, no significant differences in BMI were found in both sexes. Second, this is a cross-sectional observational study. We could not confirm a causal relationship between degenerative cervical spondylolisthesis and DCM. Third, more critical subjects might not have participated in our study; thus, selection bias is possible. Fourth, we failed to evaluate dynamic instability of the cervical spine, which should be taken into consideration in DCM evaluation.

## Conclusions

This study elucidated the prevalence of cervical spondylolisthesis according to sex, age and vertebral level and its association with DCM in a Japanese population. The prevalence of degenerative cervical posterior spondylolisthesis was increasing with age and more frequent in men than in women. Narrow canal and degenerative cervical posterior spondylolisthesis on X-ray may be useful in predicting or diagnosing DCM.

## Data Availability

All data generated or analyzed during this study are available from the corresponding author upon reasonable request.
